# Silver nanoparticles derived from* Calotropis* and* Plumeria* plants as a green approach to extend the vase life of* Alstroemeria* flowers

**DOI:** 10.1038/s41598-026-39654-6

**Published:** 2026-02-23

**Authors:** Naeimeh Abid, Davood Samsampour, Mehrdad Babarabie

**Affiliations:** 1 Horticulture Sciences Department, Faculty of Agriculture and Natural Resource, University of Hormozgan, Bandar Abbas, Iran; 2 Department of Agricultural, Minab Higher Education Complex, University of Hormozgan, Bandar Abbas, Iran

**Keywords:** Antioxidant enzymes, Peruvian lily, Plant extracts, Postharvest physiology, Tropical plants, Biochemistry, Biological techniques, Biotechnology, Nanoscience and technology, Plant sciences

## Abstract

The cut flower industry is valuable due to the aesthetic features of cut flowers; therefore, maintaining product attractiveness during the postharvest period is essential in this industry. In cut *Alstroemeria* flowers, premature yellowing of leaves on the stem before petal drop is one of the major postharvest problems, which reduces the commercial value of the flower. The experiment was conducted as a completely randomized factorial design with three replications and nine treatments at the Agricultural Faculty Laboratory of the University of Hormozgan in 2024. The treatments included chemically synthesized silver nanoparticles and green silver nanoparticles synthesized from leaf extracts of *Calotropis procera* (*Calotropis*) and *Plumeria rubra* (*Plumeria*) at concentrations of 10, 15, and 20 ml L^−1^. In addition, a 3% sucrose solution and distilled water were used as control treatments. All treatments except for the distilled water control contained 3% sucrose. The measured parameters included fresh flower weight, floret diameter, vase life, ion leakage, bud opening percentage, water uptake, total soluble solids, reducing sugars, total chlorophyll, anthocyanin, and the activities of ascorbate peroxidase and catalase enzymes. The findings clearly showed that the application of silver nanoparticles, especially those synthesized from *Plumeria* (NP) and *Calotropis* (NA) extracts, had a significant effect on improving physiological, biochemical traits, and vase life of cut *Alstroemeria* flowers. The results showed that the application of silver nanoparticles had a significant effect on improving the postharvest performance of *Alstroemeria* cut flowers. The longest vase life (18 days) and highest bud opening percentage (over 93%) were observed in the green *Plumeria* nanoparticles at 10 ml L^−1^ (NP10), while the shortest vase life (13 days) was recorded in the chemically synthesized nanoparticles at 20 ml L^−1^ (N20). The NP15 treatment increased fresh flower weight by approximately 22% and water uptake by 18% compared with the control. Moreover, the activities of ascorbate peroxidase (APX) and catalase (CAT) were 1.6‑fold and 1.4‑fold higher than those of the control, respectively. Given that biological nanoparticles appear to be more biocompatible, stable, and safer than chemical counterparts, their use in the postharvest management of ornamental flowers could potentially be a sustainable and environmentally friendly approach.

## Introduction

Over the past few decades, the cut flower industry has flourished and generated billions of dollars in the global market. Countries such as the Netherlands, China, and the United States hold significant shares in the export and trade of cut flowers worldwide^1–3^. This industry is valuable due to the aesthetic characteristics of cut flowers; therefore, maintaining the attractiveness of products during the postharvest period is essential^4^. Among cut flowers, *Alstroemeria* and its hybrids, available in various colors and forms, rank among the top 10 commercially important and popular cut flowers worldwide.

*Alstroemeria* is a perennial, rhizomatous, herbaceous plant belonging to the family Alstroemeriaceae. Native to South America, it comprises about 120 species, mostly found in Brazil and Chile^5^. Premature yellowing of leaves on the stem before petal drop is a major postharvest issue for *Alstroemeria* flowers, which reduces their commercial value^6^.

The quality of cut flowers depends on their appearance and longevity, as consumers prefer fresher flowers with longer vase life^7^. Therefore, maintaining flower quality from harvest until it reaches the consumer is a critical competitive advantage and a challenge in the cut flower supply chain^8,10^. Methods that increase vase life and preserve the quality of cut flowers are especially important. One common method to extend cut flower longevity is the use of preservative materials in vase solutions^9^.

Among various natural substances studied to improve the postharvest life of *Alstroemeria* cut flowers, silver salts—particularly silver nitrate—are widely used due to their antibacterial properties that help extend flower longevity^10^. The use of nanoparticles as antimicrobial agents to prolong cut flower life is one of the extensive agricultural applications of nanotechnology^11^. Due to their high surface-to-volume ratio, silver nanoparticles applied to cut stems not only eliminate bacteria and microorganisms in the vase solution but also enter the plant vascular tissue, inhibiting ethylene biosynthesis, preventing protein and chlorophyll degradation, improving relative water content, enhancing antioxidant activity, and ultimately extending vase life^12^.

However, determining the optimal concentration of silver nanoparticles to avoid toxic effects on cut flowers is essential, depending on factors such as nanoparticle amount, formulation type, pulse treatment duration, and cut flower species^7,13^. Langroudi et al.^14^ reported that higher concentrations of silver nanoparticles significantly increased the vase life of *Alstroemeria* flowers both before and after harvest. Researchers reported that the use of silver nanoparticles, compared with silver nitrate, increased the vase life of cut flowers and reduced microbial growth at the stem ends.

The use of preservative solutions containing chemical substances has raised concerns due to toxic effects, health risks, and environmental pollution, which has driven researchers toward natural, eco-friendly, and human-safe preservatives. Recently, plant extracts or essential oils have been employed to prolong the longevity and quality of cut flowers. Plant extracts are natural compounds with antioxidant, antifungal, and antimicrobial properties^15^, extracted from various plant organs including leaves, flowers, fruits, roots, and seeds. Since essential oils often contain phenolic compounds^16^and possess antimicrobial effects, they can eliminate bacteria in vase solutions and prevent vascular blockage^17^.

A study demonstrated that using *Calotropis procera* leaf extract as a preservative solution increased the postharvest vase life of gladiolus cut flowers, enhanced leaf water content, chlorophyll pigments, and reduced bacterial populations in the vase^7^. *Plumeria rubra*, known as *Plumeria*, is a small tree or large shrub belonging to the Apocynaceae family. More than 110 chemical constituents have been identified in various parts of *Plumeria rubra*, including iridoids, terpenoids, flavonoids and flavonoid glycosides, alkaloids, glycosides, fatty acid esters, carbohydrates, amino acids, lignans, coumarins, essential oils, and others^18^. Experimental results indicated a direct correlation between antioxidant sources and phenolic compound content; therefore, due to the antioxidant activity and presence of flavonoid and phenolic compounds in *Plumeria* leaf extract^19^, investigating the use of *Plumeria* leaf extract to improve the quality and vase life of cut *Alstroemeria* flowers is justified.

Since the use of leaf extracts from *Plumeria* and *Calotropis* plants and silver nanoparticles synthesized from them as preservative solutions for enhancing quality and postharvest longevity of cut *Alstroemeria* flowers has not been studied in domestic or international research, this study aimed to examine the response of cut *Alstroemeria* flowers to silver nanoparticles synthesized from these plants. It was hypothesized that biologically synthesized silver nanoparticles derived from *Plumeria rubra* and *Calotropis procera* would improve the postharvest quality and extend the vase life of cut *Alstroemeria* flowers by enhancing physiological and biochemical properties.

## Materials and methods

This study was conducted as a completely randomized design with a factorial arrangement, including 3 replications and 9 treatments, in the Agricultural Faculty Laboratory of Hormozgan University in 2024. The experiment began on 9 February 2024. The experimental treatments consisted of silver nanoparticles and silver nanoparticles synthesized from leaf extracts of *Calotropis procera* and *Plumeria rubra* (*Plumeria*) at concentrations of 10, 15, and 20 ml per liter, plus 3% sucrose solution and distilled water (control). Except for the control treatment (distilled water), all treatments included 3% sucrose.

To prepare the extracts, leaves of *Plumeria* and *Calotropis* were collected from the university campus. After collection and transfer to the Agricultural and Natural Resources Faculty laboratory of Hormozgan University, the leaves were separately dried in an oven at 50 °C for 24 h, then ground using a mill. For each 100 g of dried leaves, 900 ml of distilled water was added, shaken on a shaker for 4 h, and then filtered through filter paper to remove residues. The extracts were further filtered using Whatman filter paper and transferred to Falcon tubes, then centrifuged at 4000 rpm for 15 min. The supernatant was collected as the concentrated extract. To synthesize nanoparticles, 10 ml of concentrated extract was mixed with 90 ml of 1 mM silver nitrate solution and shaken for 24 h; a color change indicated nanoparticle formation^20^.

Leaves of *Calotropis procera* and *Plumeria rubra* were collected from plants located on the campus of the Faculty of Agriculture, University of Hormozgan, with the official permission of the Department of Horticultural Science. Taxonomic identification of the species was performed by Mehrdad Babarabie. Herbarium specimens of both species are deposited at the Herbarium of the Faculty of Agriculture, Minab Higher Education Complex, University of Hormozgan (UOH) under the accession numbers UOH-CP2024-01 (*Calotropis procera*) and UOH-PR2024-02 (*Plumeria rubra*).

All procedures involving the collection of plant specimens and the laboratory research conducted in this study were carried out in full compliance with all institutional, national, and international guidelines, laws, and regulations. This includes strict adherence to the policies of the University of Hormozgan and the ethical requirements of *Scientific Reports*.

*Alstroemeria* flowers of the ‘Red Sensation’ cultivar were used, purchased from a standard greenhouse in Shahreza, Isfahan. On the day of the experiment, flowers were removed from packaging, visually inspected for damage and disease, and healthy stems were cut to 30 cm length using a ruler, removing leaves up to the third node. Flowers were immediately placed in treatment solutions and stored at 9 ± 2 °C for 48 h as a pulse treatment. The weight of each flower stem was recorded individually. After 48 h, flowers were removed from treatment solutions, stems were rinsed with distilled water, and transferred to vases containing 300 ml distilled water, then stored at laboratory room temperature (24 ± 2 °C). Measurements of fresh weight, flower diameter, vase life, electrolyte leakage, bud opening rate, water uptake, total soluble solids, reducing sugars, total chlorophyll, anthocyanin content, and activities of ascorbate peroxidase and catalase enzymes were recorded on days 3, 6, and 9 of the experiment.

Fresh weight was measured with a digital scale, and flower diameter was measured with a caliper.

### Bud opening rate

The number of opened petals in treated plants was counted daily and expressed as a percentage at the end of the experiment^21^.

## Vase life

The vase life of *Alstroemeria* flowers was defined as the time when 50% of leaves turned yellow or 50% of petals had fallen; flowers were evaluated daily for these traits^22,23^.

## Enzyme activity of ascorbate peroxidase and catalase

To measure antioxidant enzyme activities, extracts were prepared. For this, 0.5 g of petals was ground with 5 ml of extraction buffer at pH 7.5 (containing 50 mM Tris buffer, 3 mM magnesium chloride, and 1 mM EDTA). The extraction buffer for ascorbate peroxidase measurement also contained 0.2 mM ascorbate. The homogenate was centrifuged at 4000 rpm for 20 min at 4 °C, and the supernatant was used as crude extract for enzyme activity assays^24^. Catalase activity was measured using Aebi’s method^25^at 240 nm, and ascorbate peroxidase activity was measured by Nakano and Asada’s method^25^ at 290 nm using a spectrophotometer.

## Water uptake (WU)

To measure this parameter, according to the method of He et al.^26^, the weight of the container holding the solution (without the flower) was measured on day zero and throughout the experiment using a scale with 0.01 g precision. The water uptake was calculated as fresh weight per gram per day (g stem^−1^ day^−1^) using the Eq. (1):

1 $$ {\text{WU }} = {\text{ }}({\mathrm{S}}_{{{\mathrm{t}} - {\mathrm{1}}}}  - {\mathrm{S}}_{{\mathrm{t}}} ) $$

Where:

WU = Water uptake (fresh weight per gram per day).

S_t−1_ = Weight of the container with solution on the current day (day 1, 3, …).

S_t_ = Weight of the container with solution on the previous day.

## Total soluble solids (TSS)

The amount of total soluble solids was measured using a digital refractometer^21^.

### Ion leakage

To determine the stability of the petal cell membranes, the electrolyte leakage index was used. The method of Sairam and Srivastava^27^was applied, and readings were taken using an EC (electrical conductivity) meter. Approximately one gram of petals was placed in a 15 ml Falcon tube, then 10 ml of distilled water was added. The samples were kept at room temperature for 24 h and then the initial electrical conductivity (EC1) was measured. After sealing the Falcon tubes, they were autoclaved at 120 °C for 20 min to kill the cells, then cooled down and the secondary electrical conductivity (EC2) was measured. Electrolyte leakage was calculated using Eq. (2):

2 $${\text{El }}\left( {{\mathrm{mS}}} \right){\text{ }}={\text{ }}\{ ({\mathrm{EC2}}\, - \,{\mathrm{EC1}})/{\mathrm{EC2}}\}  \times {\mathrm{1}}00$$

Where:

EL = Electrolyte leakage index.

EC1 = Initial electrical conductivity.

EC2 = Secondary electrical conductivity.

## Total chlorophyll

Total chlorophyll content was measured according to Lichtenthaler’s method^28^. Leaf samples were collected on days 3, 6, and 9 from treated plants. To determine chlorophyll content, 0.5 g of leaf tissue was ground in liquid nitrogen using a mortar and pestle and placed in a test tube. Then, 5 ml of pure methanol (100%) was added to the leaf sample. To prevent photodegradation of chlorophyll and dissolving of photosynthetic pigments in the solvent, samples were kept in the dark for 24 h. After centrifugation, the supernatant was used to measure absorbance at wavelengths 652 nm and 665 nm using a spectrophotometer. Chlorophyll content was calculated using Eq. (3):

3 $$\begin{gathered}  {\text{Chlorophyll a}}={\text{ }}\left( {{\mathrm{16}}.{\text{72 }}{{\mathrm{A}}_{{\mathrm{665}}}}--{\text{ 9}}.{\text{16 }}{{\mathrm{A}}_{{\mathrm{652}}}}} \right){\text{ }} \times {\text{ }}\left( {{\mathrm{v}}/{\mathrm{w}}} \right) \hfill \\  {\text{Cholorophyll b }}={\text{ }}\left( {{\mathrm{36}}.{\mathrm{92}}{{\mathrm{A}}_{{\mathrm{652}}}}--{\text{ 16}}.{\text{54 }}{{\mathrm{A}}_{{\mathrm{665}}}}} \right){\text{ }} \times {\text{ }}\left( {{\mathrm{v}}/{\mathrm{w}}} \right) \hfill \\  {\text{Total chlorophyll}}={\text{ Chlorophyll a}}\,+\,{\text{Cholorophyll b}} \hfill \\  {\mathrm{A665}}\,=\,{\text{Absorbance at 665 nm}} \hfill \\  {\mathrm{A652A}}\,=\,{\text{Absorbance at 652 nm}} \hfill \\  {\mathrm{V}}\,=\,{\text{Volume of methanol }}\left( {{\mathrm{ml}}} \right) \hfill \\  {\mathrm{W}}\,=\,{\text{Weight of ground tissue }}\left( {\mathrm{g}} \right) \hfill \\ \end{gathered} $$

## Petal anthocyanin

Petal anthocyanin was measured using the Wagner^29^ method. For this purpose, 0.1 g of fresh petal tissue from each sample was ground with 10 ml of acidic methanol (methanol containing 1% HCl). The resulting extracts were centrifuged at 6000 rpm for 10 min. Then, the supernatant solutions were kept in the dark at room temperature for 24 h. After 24 h, the absorbance of each sample was read at 550 nm using a spectrophotometer. To calculate the anthocyanin concentration, an extinction coefficient of 33,000 mM^−1^ cm^−1^ was used.

### Reducing sugar

Reducing sugar was measured using the Somogyi method^30^. For this, 0.02 g of petal tissue was ground with 10 ml of methanol in a mortar and then transferred to a beaker, which was heated until boiling. Once boiling point was reached, heating was stopped and the contents were filtered through filter paper to obtain the plant extract. 2 ml of each extract were transferred into test tubes, followed by the addition of 2 ml of copper sulfate solution. The tubes were then placed in a water bath at 100 °C for 20 min. During this process, Cu²⁺ ions were reduced by monosaccharide aldehydes to Cu_2_O, which appeared as a brick-red precipitate at the bottom of the tubes. After cooling, 2 ml of phosphomolybdic acid was added, resulting in a blue coloration after a few minutes. The test tubes were vigorously shaken to evenly distribute the blue color throughout. Then, absorbance was read at 600 nm by a spectrophotometer, and the concentration of reducing sugars was calculated based on a standard curve, expressed in mg/g fresh weight.

### Characterization and measurement of biosynthesized silver nanoparticles

The characterization analyses of the synthesized silver nanoparticles, including measurement of zeta potential, UV-Vis spectrum, and particle size (TEM), were performed at the Central Laboratory of the University of Urmia (Urmia, Iran).

**Fig. 1:**
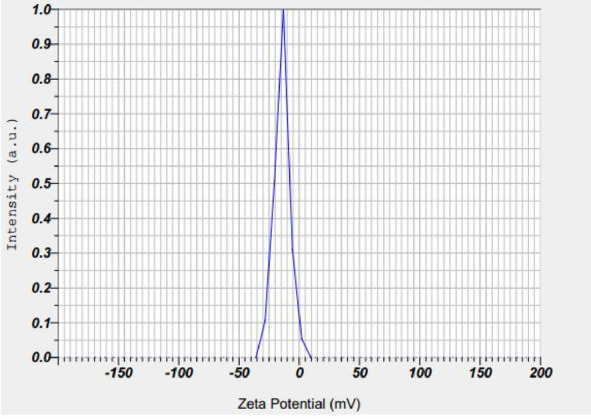
Zeta potential of *Plumeria rubra* silver nanoparticles.

**Fig. 2:**
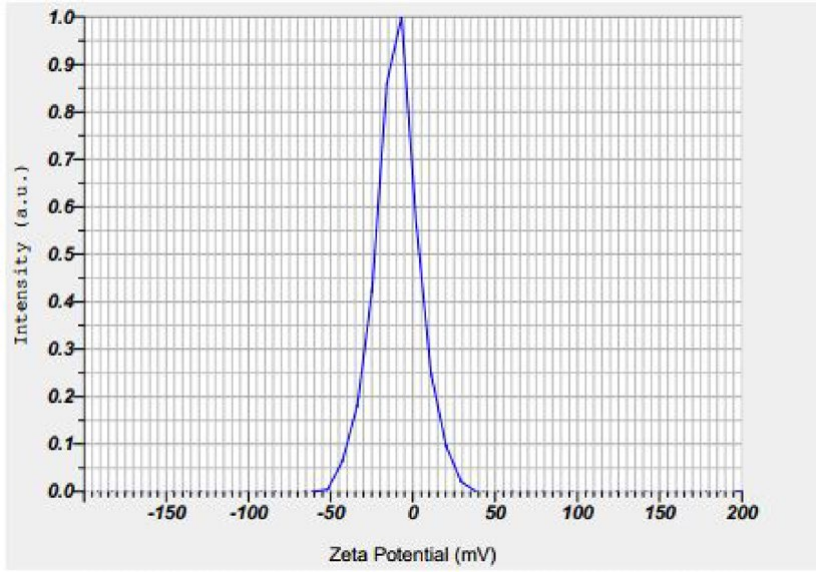
Zeta potential of *Calotropis procera* silver nanoparticles.

The stability of the silver nanoparticles synthesized using extracts of *Plumeria rubra* and *Calotropis procera* was evaluated based on their zeta potential values. The zeta potential of *P. rubra*-derived nanoparticles was approximately − 28 mV (Fig. 1), and that of *C. procera*-derived nanoparticles was around − 32 mV (Fig. 2), indicating good colloidal stability of the suspensions.

The UV–visible absorption spectra of biosynthesized silver nanoparticles from *Plumeria rubra* and *Calotropis procera* extracts exhibited a strong surface plasmon resonance (SPR) peak around 430 nm, confirming the formation of silver nanoparticles.

**Fig. 3:**
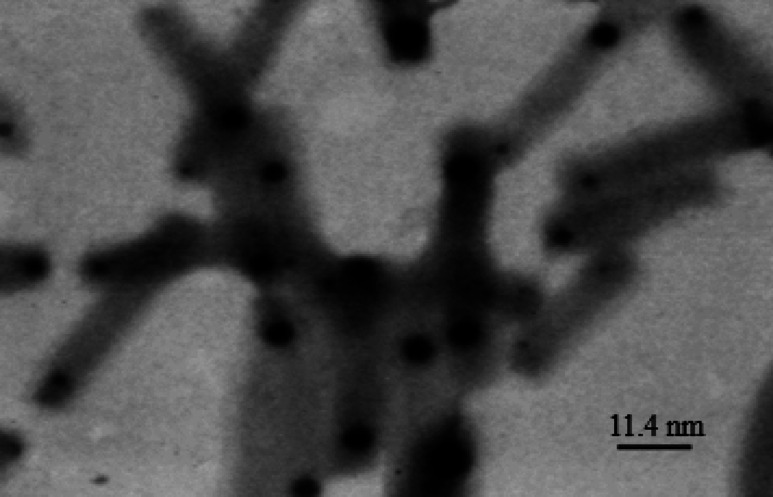
TEM image of silver nanoparticles synthesized with *Plumeria rubra* extract (average size: 11.4 nm).

**Fig. 4:**
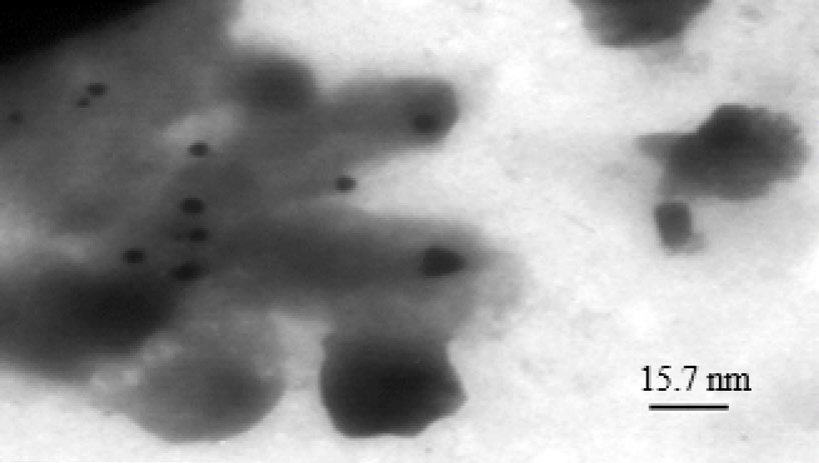
TEM image of silver nanoparticles synthesized with *Calotropis procera* extract (average size: 15.7 nm).

TEM images of *P. rubra* (Fig. 3) and *C. procera* (Fig. 4) silver nanoparticles revealed particles with mean sizes of 11.4 nm and 15.7 nm, respectively, which were agglomerated and uniformly dispersed within an organic matrix.

### Data analysis

Data were analyzed using SAS statistical software. Prior to the analysis of variance (ANOVA), the assumptions of normality and homogeneity of variances were confirmed. Means were then compared using the LSD test at a 5% significance level.

## Results

The results of variance analysis showed that the individual effects of treatment, time, and their interaction on traits (flower diameter, water uptake, ion leakage, fresh flower weight, total chlorophyll, soluble solids, reducing sugar, anthocyanin, and the enzymes ascorbate peroxidase and catalase) were significant at the 1% level (Tables 1 and 2). Additionally, the effect of treatments on the traits of vase life and the percentage of flower opening in cut *Alstroemeria* flowers was significant at the 1% level (Table 3).

**Table 1:** Effects of treatments, time, and their interaction on measured traits of cut *Alstroemeria* flowers.

S.O.V	df	Ascorbate Peroxidase (µmol H_2_O_2_ min^−1^ mg^−1^ protein)	Catalase (µmol H_2_O_2_ min^−1^ mg^−1^ protein)	Anthocyanin (mg g^−1^ FW)	Reducing Sugar (mg g^−1^ FW)	Soluble Solids (Brix)
Treatment	10	0.018**	0.005**	0.005**	0.009**	0.561**
Time	2	1.602**	0.326**	0.010**	0.003**	0.462**
Treatment * Time	20	0.008**	0.006**	0.003**	0.009*	0.594**
Error	64	0.0009	0.0001	0.00003	0.0003	0.146
CV	–	5.055	4.840	4.578	6.311	6.959

*, **indicates a significant difference at *P* < 0.05 & *P* < 0.01 respectly.

**Table 2:** Effects of treatments, time, and their interaction on measured traits of cut *Alstroemeria* flowers.

S.O.V	df	Ion Leakage (%)	Petal Diameter (mm)	Water Absorption (ml^− 1^)	Total Chlorophyll (mg g^−1^ FW)	Fresh Weight of Flower (g )
Treatment	10	115.795**	194.691**	96.891**	0.017**	528.654**
Time	2	81.532**	3492.132**	6305.727**	0.478**	16540.636**
Treatment * Time	20	57.473**	151.287**	59.827**	0.022**	30.136**
Error	64	0.371	0.438	0.827	0.00005	0.527
CV	–	1.554	1.157	3.542	1.799	1.949

**indicates a significant difference at *P* < 0.01.

**Table 3:** Effects of treatments on measured traits of cut *Alstroemeria* flowers.

S.O.V	df	Bud Opening Percentage (%)	Vase Life (days)
Treatment	10	174.91**	8.26**
Block	2	1.07^ns^	0.0003^ns^
Error	20	3.15	0.30
CV	–	2.21	3.68

**indicates a significant difference at *P* < 0.01; ns , not significant.

### Vase life and percentage of flower bud opening

**Fig. 5:**
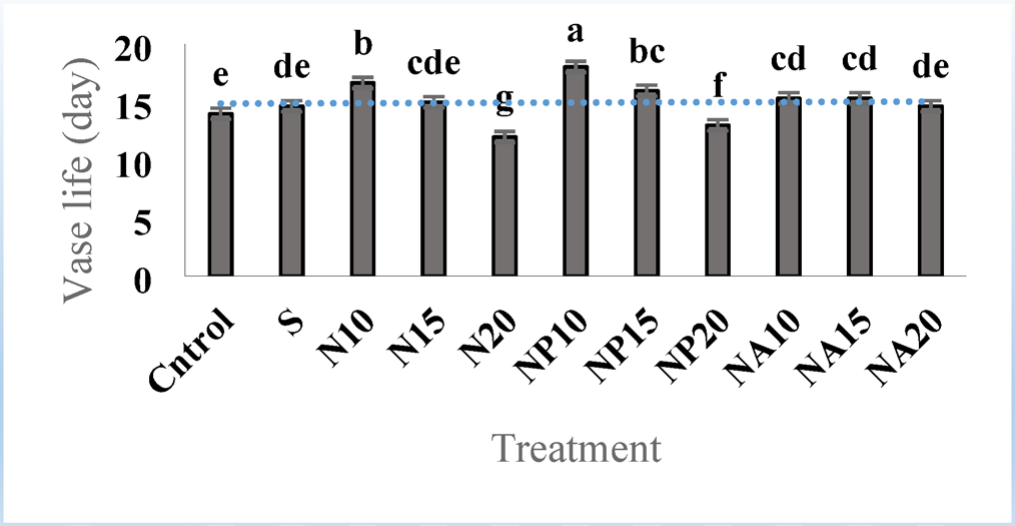
Mean comparison of the effects of sucrose, chemically synthesized silver nanoparticles, and plant-synthesized silver nanoparticles on the vase life of cut *Alstroemeria* flowers. C,  Control (distilled water): S,  Sucrose 3%; N10, N15: N20 ,  Chemically synthesized silver nanoparticles at 10, 15, and 20 mg L^−1^; NP10, NP15: NP20, Biologically synthesized silver nanoparticles using *Plumeria rubra* leaf extract at 10, 15, and 20 ml L^−1^; NA10, NA15: NA20, Biologically synthesized silver nanoparticles using *Calotropis procera* leaf extract at 10, 15, and 20 ml L^−1^.

According to Fig. 5, the longest vase life (18 days) was related to the NP10 treatment, and the shortest vase life (13 days) was related to the N20 treatment. The present study shows that increasing the concentration of green *Plumeria* nanoparticles beyond 10 milliliters per liter does not increase flower longevity. Also, green silver nanoparticles at concentrations of 10 and 15 milliliters per liter increased vase life compared to the control, although at 20 milliliters per liter there was no significant difference compared to the control.

**Fig. 6:**
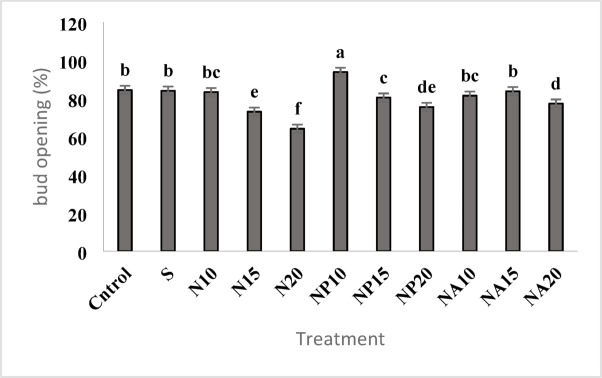
Mean comparison of the effects of sucrose, chemically synthesized silver nanoparticles, and plant-synthesized silver nanoparticles on the bud opening percentage of cut *Alstroemeria* flowers. C,  Control (distilled water): S, Sucrose 3%; N10, N15: N20, Chemically synthesized silver nanoparticles at 10, 15, and 20 mg L^−1^; NP10, NP15: NP20, Biologically synthesized silver nanoparticles using *Plumeria rubra* leaf extract at 10, 15, and 20 ml L^−1^; NA10, NA15: NA20 ,  Biologically synthesized silver nanoparticles using *Calotropis procera* leaf extract at 10, 15, and 20 ml L^−1^.

According to Fig. 6, the highest percentage of flower bud opening was recorded in the NP10 treatment with an average of over 93%, and the lowest opening percentage was in the N20 treatment with an average of about 65%.

### Flower diameter

**Fig. 7:**
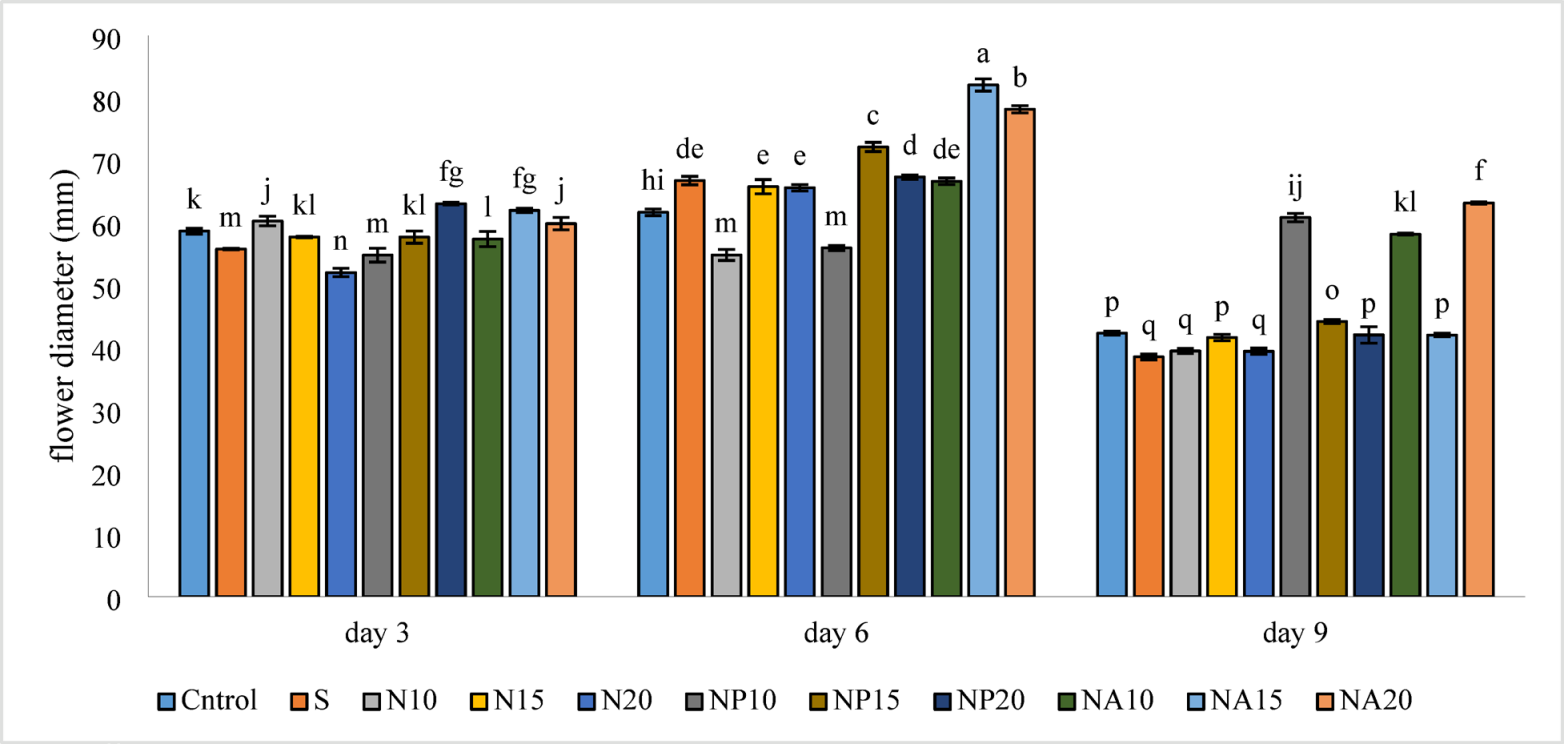
Mean comparison of the interaction effects of sucrose, chemically synthesized silver nanoparticles, and biologically synthesized silver nanoparticles over time on the amount of bud diameter of cut *Alstroemeria* flowers. C,  Control (distilled water): S,  Sucrose 3%; N10, N15: N20, Chemically synthesized silver nanoparticles at 10, 15, and 20 mg L^−1^; NP10, NP15: NP20 , Biologically synthesized silver nanoparticles using *Plumeria rubra* leaf extract at 10, 15, and 20 ml L^−1^; NA10, NA15: NA20, Biologically synthesized silver nanoparticles using *Calotropis procera* leaf extract at 10, 15, and 20 ml L^−1^.

The examination of changes in flower bud diameter showed that different treatments had a significant effect on this trait (Fig. 7). The largest flower diameter was observed in the NA15 treatment (82.1 mm) on the sixth day, and the smallest flower diameter was related to the (S, N10) treatments (38.46, 39.42 mm, respectively) on the ninth day.

### Water uptake

**Fig. 8:**
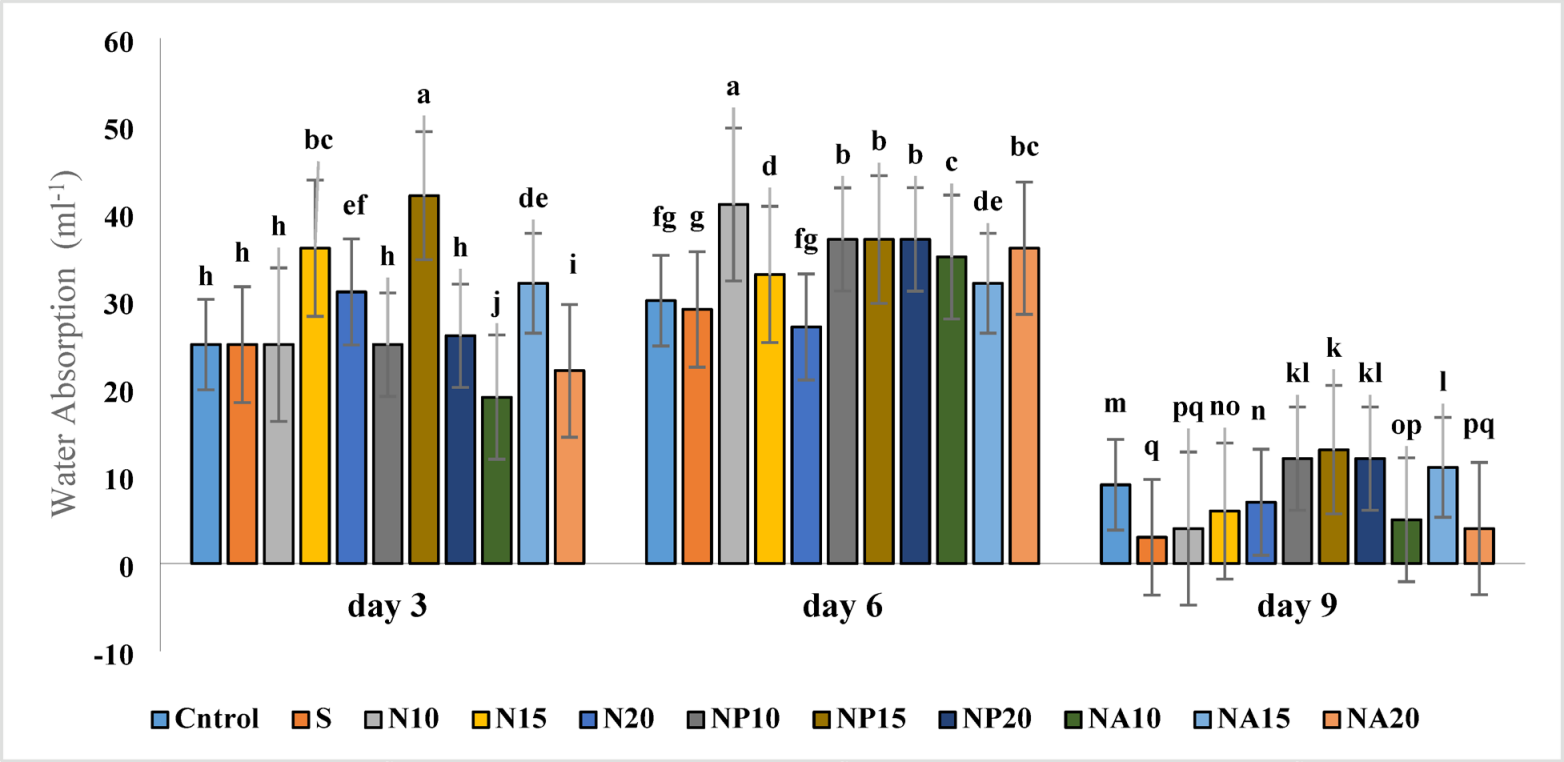
Mean comparison of the interaction effects of sucrose, chemically synthesized silver nanoparticles, and biologically synthesized silver nanoparticles over time on the amount of water uptake of cut *Alstroemeria* flowers. C,  Control (distilled water): S , Sucrose 3%; N10, N15: N20, Chemically synthesized silver nanoparticles at 10, 15, and 20 mg L^−1^; NP10, NP15: NP20, Biologically synthesized silver nanoparticles using *Plumeria rubra* leaf extract at 10, 15, and 20 ml L^−1^; NA10, NA15: NA20, Biologically synthesized silver nanoparticles using *Calotropis procera* leaf extract at 10, 15, and 20 ml L^−1^.

The examination of water uptake in flowers showed that the highest water uptake was related to the N10 treatment (41 ml^− 1^) on the third day and the N10 treatment on the sixth day, which were significantly higher than other treatments. The lowest water uptake was observed in the sucrose (S) treatment (3 ml^− 1^) on the ninth day (Fig. 8). Overall, silver nanoparticles synthesized with *Plumeria* extract (NP), especially at medium to high concentrations (NP15 and NP20), had the most positive effect on water uptake, likely related to improved physiological traits of the flowers such as stomatal opening and better absorption through the stem.

### Fresh flower weight

**Fig. 9:**
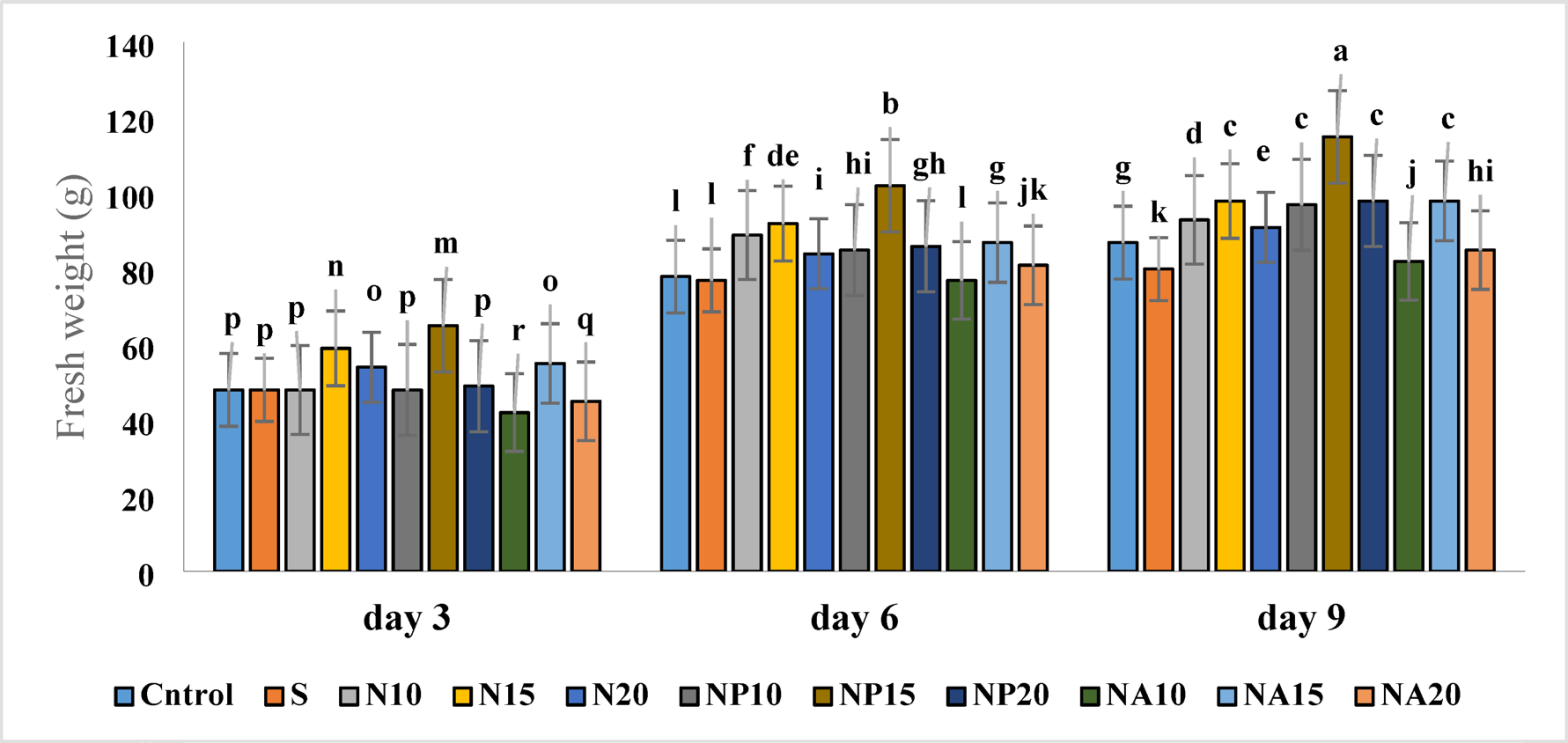
Mean comparison of the interaction effects of sucrose, chemically synthesized silver nanoparticles, and biologically synthesized silver nanoparticles over time on the amount of fresh weight of cut *Alstroemeria* flowers. C,  Control (distilled water): S,  Sucrose 3%; N10, N15: N20,  Chemically synthesized silver nanoparticles at 10, 15, and 20 mg L^−1^; NP10, NP15: NP20,  Biologically synthesized silver nanoparticles using *Plumeria rubra* leaf extract at 10, 15, and 20 ml L^−1^; NA10, NA15: NA20, Biologically synthesized silver nanoparticles using *Calotropis procera* leaf extract at 10, 15, and 20 ml L^−1^.

The investigation of the effects of different treatments on fresh flower weight showed that the highest fresh weight was related to the NP15 treatment (115.05 g) on the ninth day, and the lowest amount was related to the NA10 treatment (42.05 g) on the third day (Fig. 9).

### Soluble solids (TSS)

**Fig. 10:**
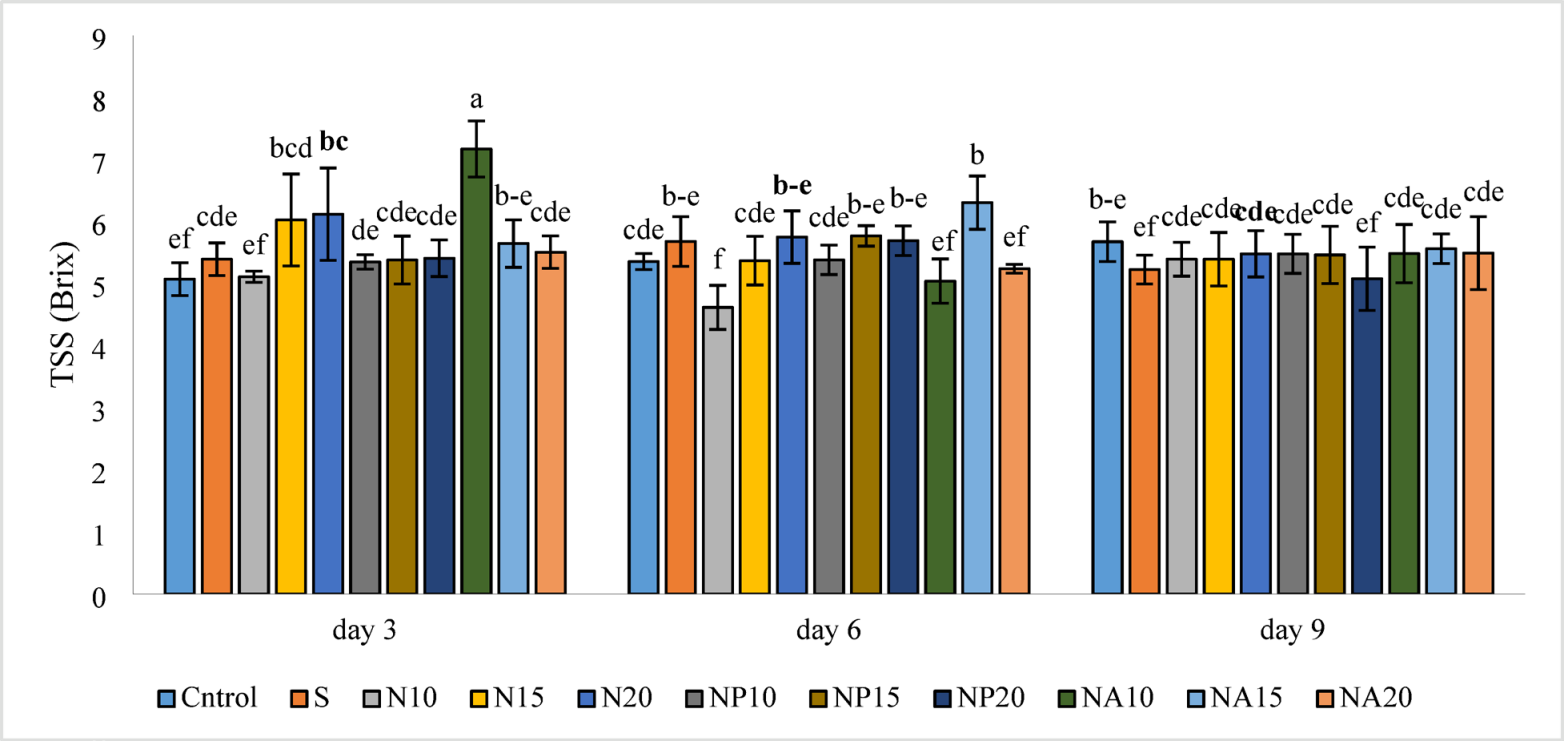
Mean comparison of the interaction effects of sucrose, chemically synthesized silver nanoparticles, and biologically synthesized silver nanoparticles over time on the amount of TSS of cut *Alstroemeria* flowers. C,  Control (distilled water): S, Sucrose 3%; N10, N15: N20,  Chemically synthesized silver nanoparticles at 10, 15, and 20 mg L^−1^; NP10, NP15: NP20, Biologically synthesized silver nanoparticles using *Plumeria rubra* leaf extract at 10, 15, and 20 ml L^−1^; NA10, NA15: NA20, Biologically synthesized silver nanoparticles using *Calotropis procera* leaf extract at 10, 15, and 20 ml L^−1^.

According to Fig. 10, the highest amount of soluble solids was observed on the third day in the NA10 treatment (7.17 brix), and the lowest amount was related to the N10 treatment (4.62 brix) on the sixth day.

### Ion leakage

**Fig. 11:**
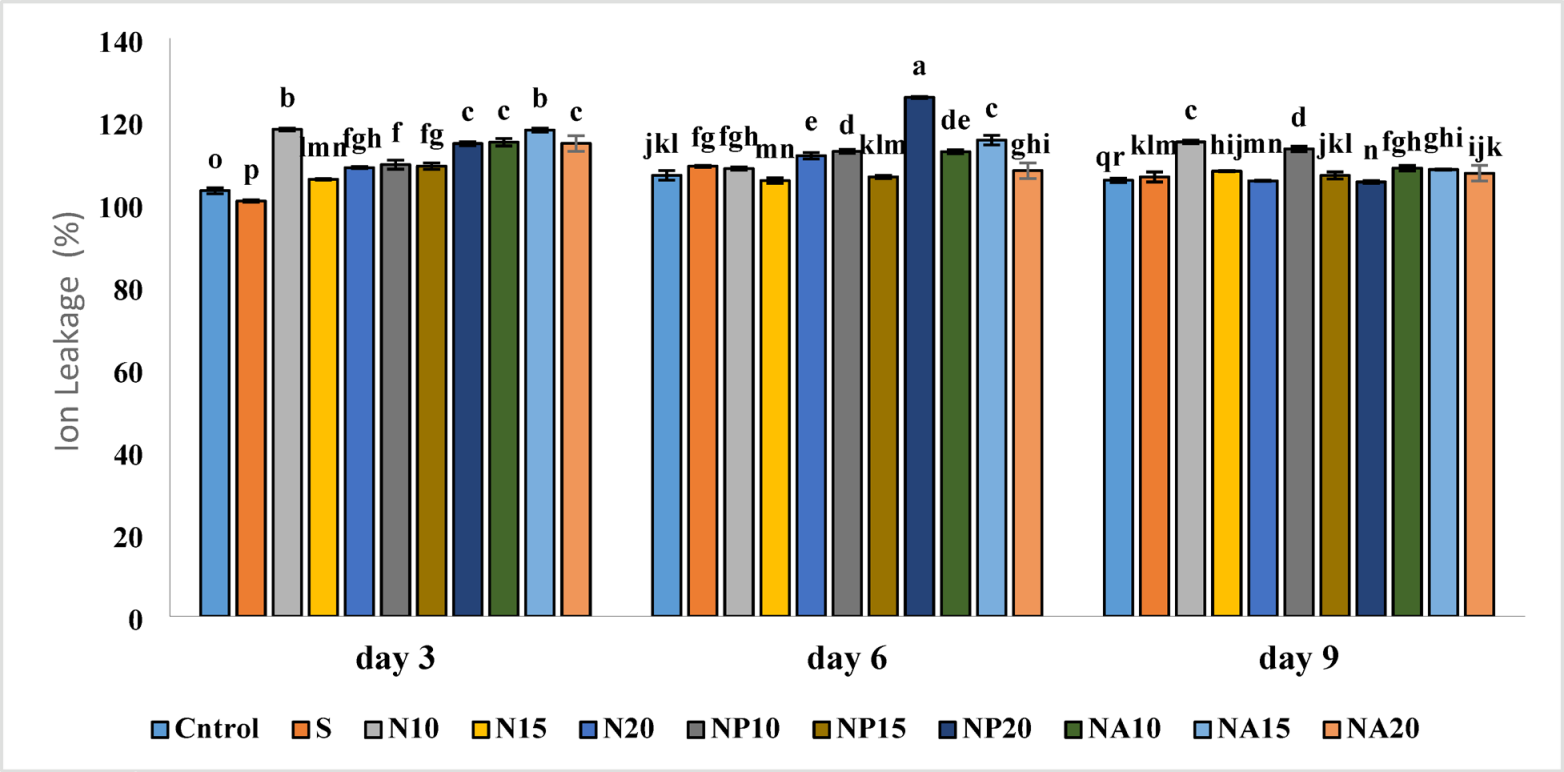
Mean comparison of the interaction effects of sucrose, chemically synthesized silver nanoparticles, and biologically synthesized silver nanoparticles over time on the amount of ion leakage of cut *Alstroemeria* flowers. C,  Control (distilled water); S,  Sucrose 3%; N10, N15: N20, Chemically synthesized silver nanoparticles at 10, 15, and 20 mg L^−1^; NP10, NP15: NP20, Biologically synthesized silver nanoparticles using *Plumeria rubra* leaf extract at 10, 15, and 20 ml L^−1^; NA10, NA15: NA20, Biologically synthesized silver nanoparticles using *Calotropis procera* leaf extract at 10, 15, and 20 ml L^−1^.

Results related to leaf ion leakage showed that the highest ion leakage occurred in the NP20 treatment (125.75%) on the sixth day, followed by the N10 and NA15 treatments on the third day, while the lowest ion leakage was observed on the third day in the sucrose (S) treatment (100.61%) (Fig. 11).

### Total chlorophyll

**Fig. 12:**
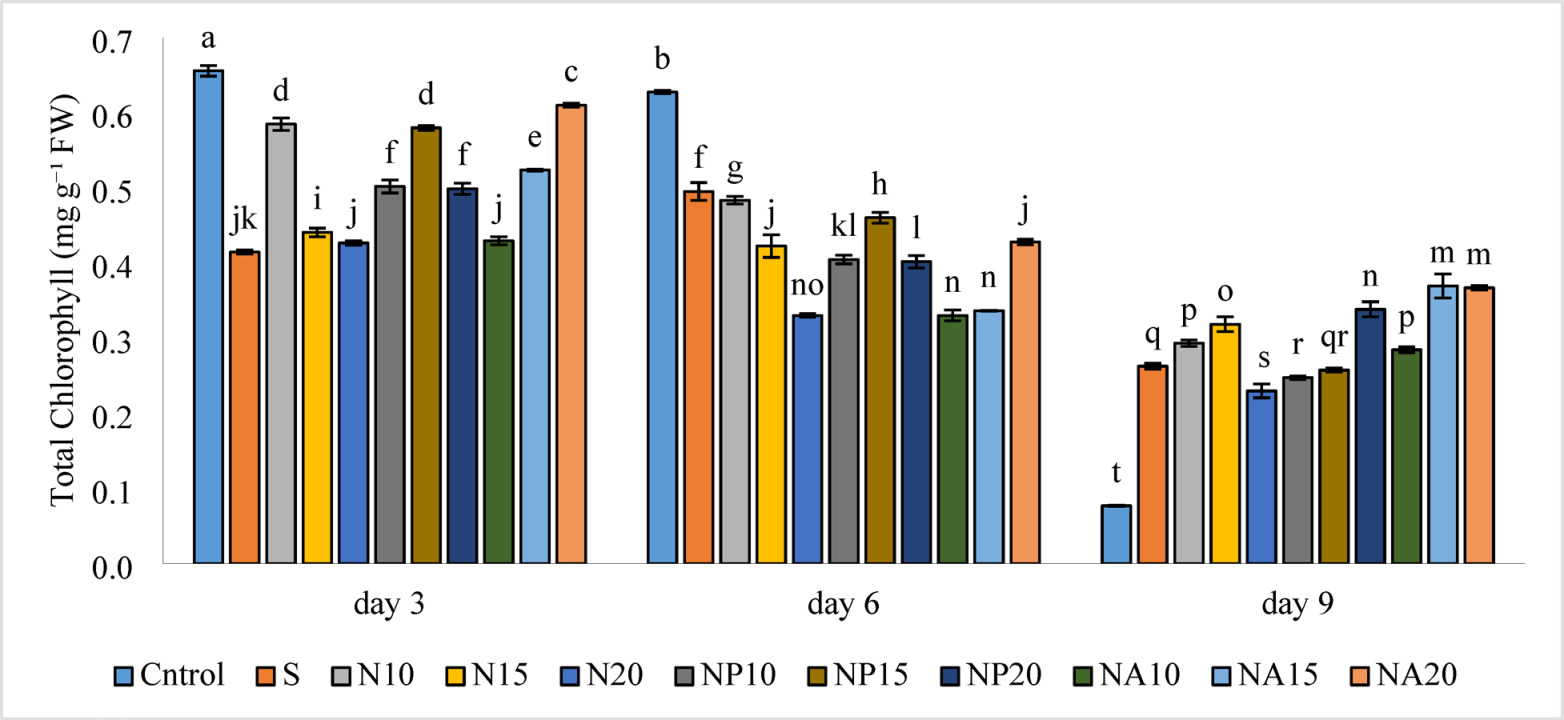
Mean comparison of the interaction effects of sucrose, chemically synthesized silver nanoparticles, and biologically synthesized silver nanoparticles over time on the amount of total cholorophyll of cut *Alstroemeria* flowers. C,  Control (distilled water); S = Sucrose 3%; N10, N15: N20, Chemically synthesized silver nanoparticles at 10, 15, and 20 mg L^−1^; NP10, NP15: NP20, Biologically synthesized silver nanoparticles using *Plumeria rubra* leaf extract at 10, 15, and 20 ml L^−1^; NA10, NA15: NA20, Biologically synthesized silver nanoparticles using *Calotropis procera* leaf extract at 10, 15, and 20 ml L^−1^.

According to Fig. 12, the highest total chlorophyll content was observed in the control treatment (0.66 mg g^−1^ FW) on the third day, and the lowest chlorophyll content in the same treatment (0.08 mg g^−1^ FW) was recorded on the ninth day.

### Anthocyanin

**Fig. 13:**
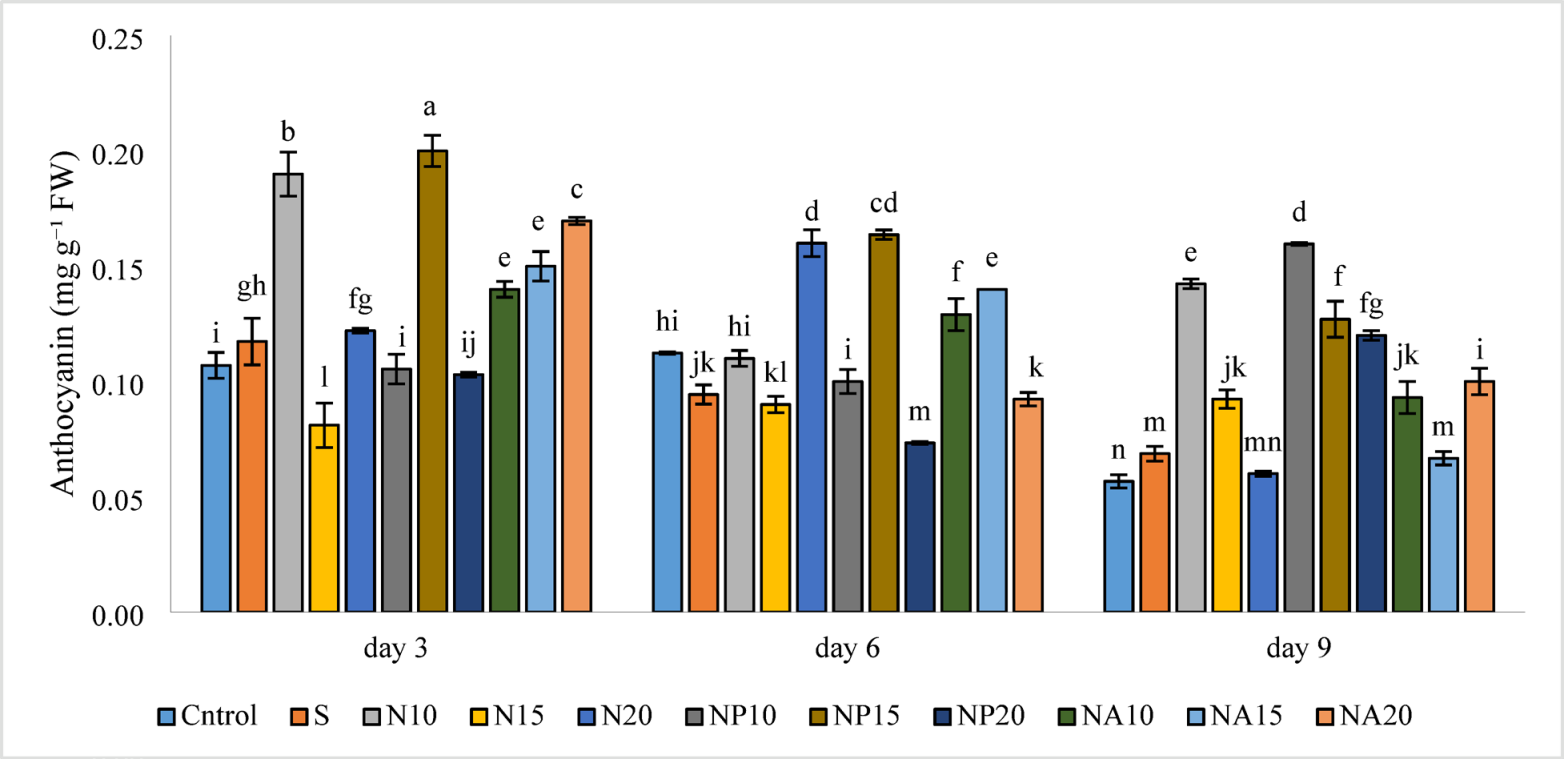
Mean comparison of the interaction effects of sucrose, chemically synthesized silver nanoparticles, and biologically synthesized silver nanoparticles over time on the anthocyanin capacity of cut *Alstroemeria* flowers. C,  Control (distilled water): S, Sucrose 3%; N10, N15: N20,  Chemically synthesized silver nanoparticles at 10, 15, and 20 mg L^−1^; NP10, NP15: NP20, Biologically synthesized silver nanoparticles using *Plumeria rubra* leaf extract at 10, 15, and 20 ml L^−1^; NA10, NA15: NA20, Biologically synthesized silver nanoparticles using *Calotropis procera* leaf extract at 10, 15, and 20 ml L^−1^.

Results from the assessment of anthocyanin content in cut *Alstroemeria* flowers, shown in Fig. 13, indicate that the NP15 treatment produced the highest anthocyanin level (0.20 mg g^−1^ FW) on the third day among all treatments, while the lowest amount was observed in the control treatment (0.06 mg g^−1^ FW) on the ninth day, with no significant difference compared to the N20 treatment (0.06 mg g^−1^ FW) on the ninth day.

### Reducing sugars

**Fig. 14:**
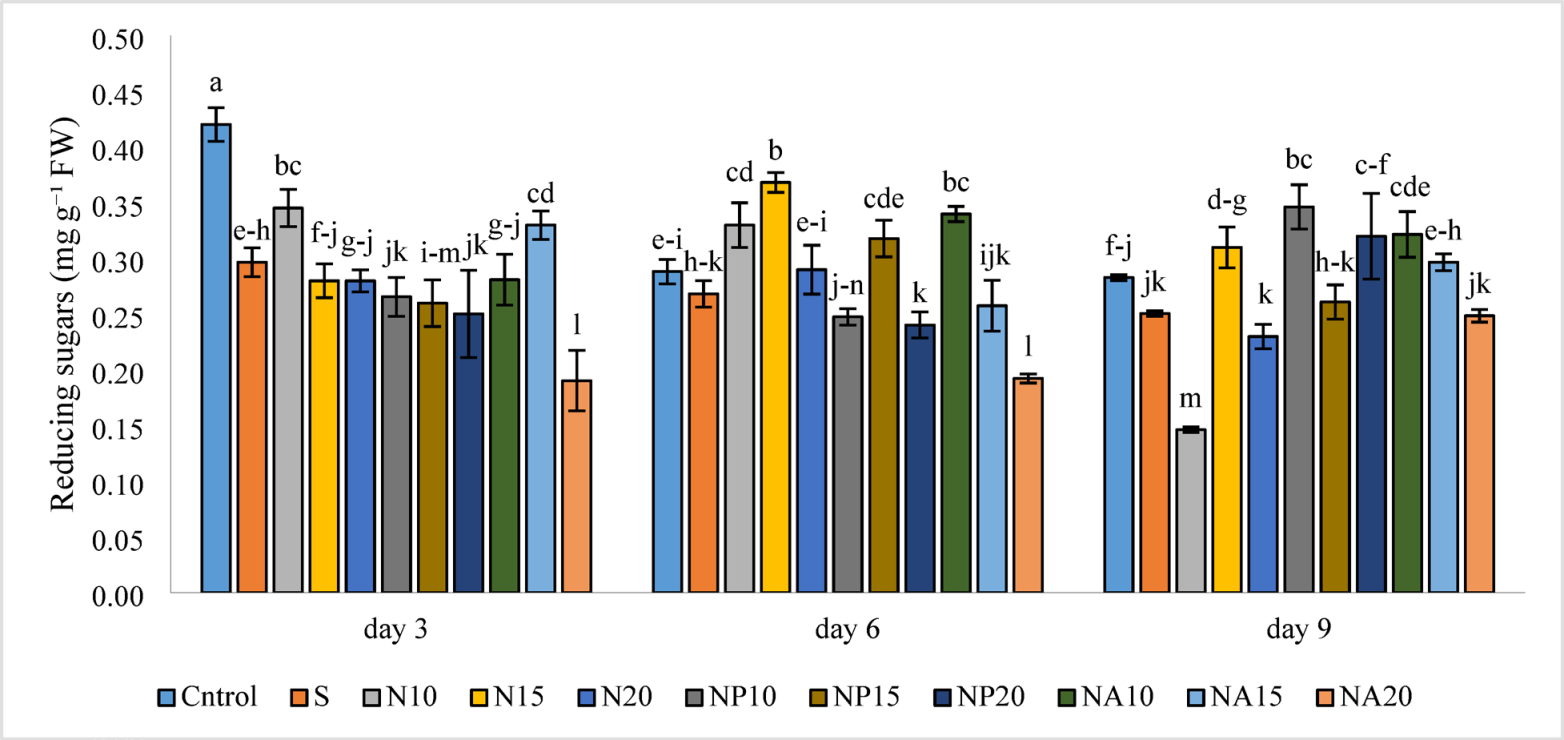
Mean comparison of the interaction effects of sucrose, chemically synthesized silver nanoparticles, and biologically synthesized silver nanoparticles over time on the reducing sugars of cut *Alstroemeria* flowers. C, Control (distilled water): S,  Sucrose 3%; N10, N15: N20, Chemically synthesized silver nanoparticles at 10, 15, and 20 mg L^−1^; NP10, NP15: NP20, Biologically synthesized silver nanoparticles using *Plumeria rubra* leaf extract at 10, 15, and 20 ml L^−1^; NA10, NA15: NA20, Biologically synthesized silver nanoparticles using *Calotropis procera* leaf extract at 10, 15, and 20 ml L^−1^.

Based on Fig. 14, the results of examining changes in reducing sugars under different treatments showed that the highest amount of reducing sugars was observed on the third day in the control treatment (0.42 mg g^−1^ FW), and the lowest amount was observed on the ninth day in the N10 treatment (0.15 mg g^−1^ FW). On the sixth day, the N15 treatment showed the highest reducing sugar content, likely due to stimulation of metabolic pathways under the influence of chemical nanoparticles. On the same day, the NA10 treatment also exhibited high levels of reducing sugars, indicating the role of low concentrations of nanoparticles in enhancing physiological activities of the plant.

### Activity of ascorbate peroxidase and catalase enzymes

**Fig. 15:**
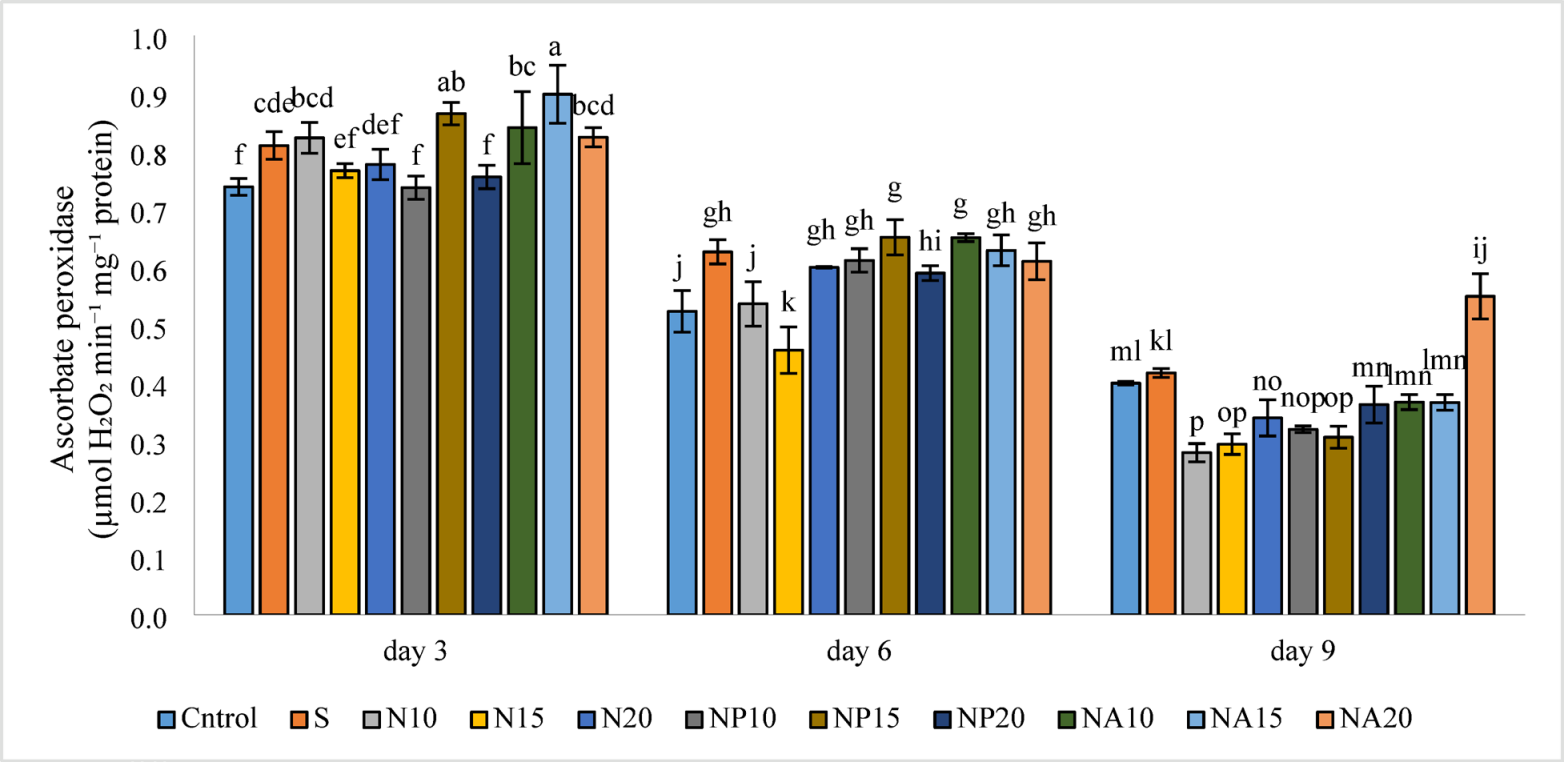
Mean comparison of the interaction effects of sucrose, chemically synthesized silver nanoparticles, and biologically synthesized silver nanoparticles over time on the ascorbate peroxidase enzyme activity of cut *Alstroemeria* flowers. C, Control (distilled water): S,  Sucrose 3%; N10, N15: N20, Chemically synthesized silver nanoparticles at 10, 15, and 20 mg L^−1^; NP10, NP15: NP20,  Biologically synthesized silver nanoparticles using *Plumeria rubra* leaf extract at 10, 15, and 20 ml L^−1^; NA10, NA15: NA20, Biologically synthesized silver nanoparticles using *Calotropis procera* leaf extract at 10, 15, and 20 ml L^−1^.

**Fig. 16:**
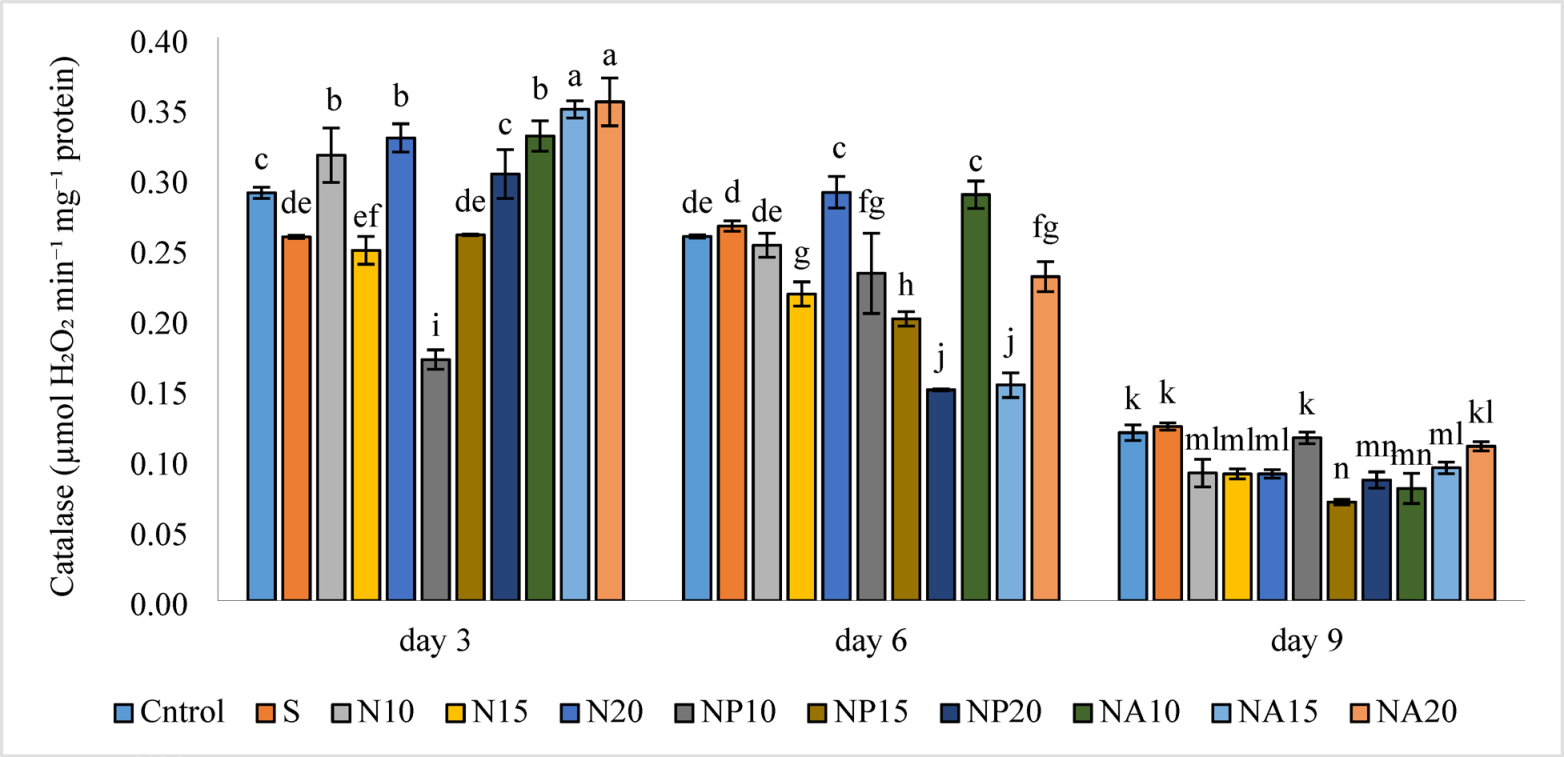
Mean comparison of the interaction effects of sucrose, chemically synthesized silver nanoparticles, and biologically synthesized silver nanoparticles over time on the catalase enzyme activity of cut *Alstroemeria* flowers. C,  Control (distilled water): S, Sucrose 3%; N10, N15: N20, Chemically synthesized silver nanoparticles at 10, 15, and 20 mg L^−1^; NP10, NP15: NP20, Biologically synthesized silver nanoparticles using *Plumeria rubra* leaf extract at 10, 15, and 20 ml L^−1^; NA10, NA15: NA20, Biologically synthesized silver nanoparticles using *Calotropis procera* leaf extract at 10, 15, and 20 ml L^−1^.

Results in Fig. 15 show that the highest activity of ascorbate peroxidase enzyme was observed in the NA15 treatment (0.90 µmol) on day three, which was not significantly different from the NP15 (0.87 µmol) treatment. This indicates that biologically synthesized silver nanoparticles with plant extracts were able to protect the flower’s antioxidant defense system against aging. The lowest activity of this enzyme was related to the N10 treatment (0.28 µmol) on day nine, which showed no significant difference compared to treatments N15, NP10, and NP15. Figure 16 shows that the highest catalase enzyme activity occurred on day three in treatments NA15 and NA20 (0.35 µmol), which were significantly higher than other treatments. Meanwhile, the lowest catalase activity was observed on day nine in the NP15 treatment (0.07 µmol), which was not significantly different from NP20 and NA10 treatments.

#### Results of correlations between measured traits

**Fig. 17:**
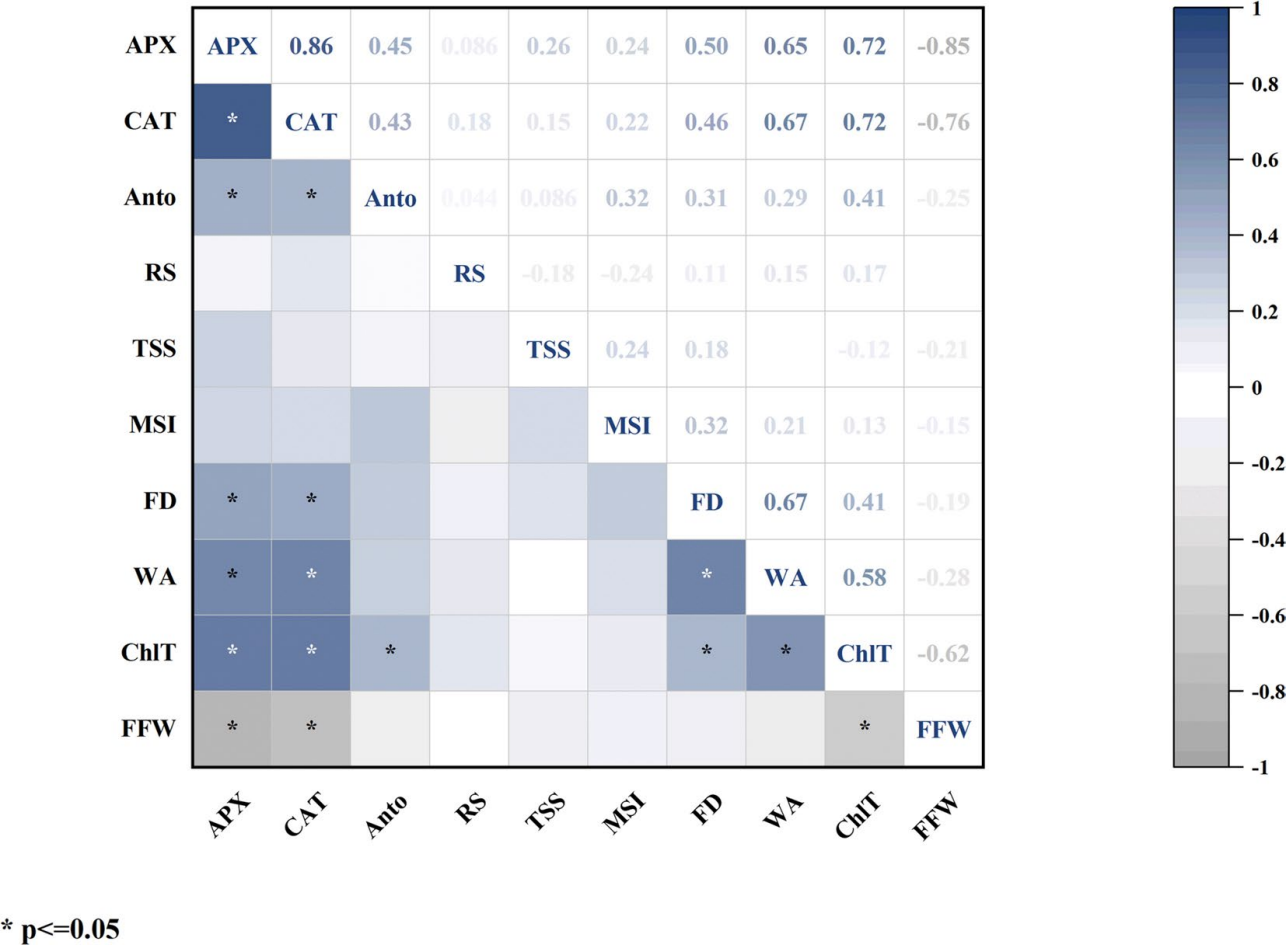
Heatmap showing the correlation among measured traits in *Alstroemeria* flowers in response to chemically synthesized and plant-synthesized silver nanoparticles and sucrose.

The correlation map (Fig. 17) illustrates the relationships between various physiological and biochemical traits across different nanoparticle treatments. Darker colors indicate stronger correlations, and asterisks (*) denote statistical significance at the 5% level.

A very strong and significant positive correlation was observed between ascorbate peroxidase and catalase enzymes (*r* = 0.86, *P* < 0.01), indicating that the activities of these two antioxidant enzymes are closely aligned and increase together in response to stresses or stimuli from nanoparticle treatments. Such synergistic behavior is commonly reported in studies dealing with oxidative stress defense.

Water content of the flower (WA) also showed a significant positive correlation with both ascorbate peroxidase (**r = 0.65*) and catalase (**r = 0.67*), reflecting the relationship between flower hydration status and antioxidant capacity. Treatments that enhanced antioxidant activity prevented water loss and improved cellular stability.

Total chlorophyll was positively and significantly correlated with both ascorbate peroxidase (**r = 0.71*) and catalase (**r = 0.72*), indicating that strengthening the antioxidant system also helps maintain photosynthetic structures, keeping chlorophyll levels higher.

Flower diameter exhibited significant positive correlations with ascorbate peroxidase (**r = 0.50*), catalase (**r = 0.46*), and water content (**r = 0.67*), suggesting that flowers with better antioxidant enzyme activity and hydration also had improved flower size. Conversely, fresh weight of the flower showed a significant negative correlation with ascorbate peroxidase (**r = − 0.85*) and catalase (**r = − 0.75**). This may indicate that increased antioxidant activity in some treatments favored maintaining cellular quality and longevity rather than merely increasing the wet mass of the flower.

Soluble solids content showed no significant correlations with other traits (–0.21 ≤ *r* ≤ 0.19) and appeared to act independently, possibly due to its different metabolic pathway or a direct effect of sucrose as a precursor. Reducing sugars also lacked significant correlations with other traits (0.10 ≤ *r* ≤ 0.16), likely because of their more independent variations and lesser dependence on physiological and enzymatic traits.

Overall, the heatmap provides a detailed visualization of key trait interrelations, demonstrating that treatments increasing antioxidant enzyme activities simultaneously improved hydration status, chlorophyll retention, and flower size. Meanwhile, some traits like fresh weight showed contrasting trends (*r* = − 0.62 with ChlT), possibly reflecting a balance between growth and longevity.

As evidenced by the obtained data, the superiority of NP15 and NA15 treatments in most physiological and biochemical characteristics (including fresh weight, chlorophyll content, anthocyanin, reducing sugars, and vase life) indicates the synergistic performance of green silver nanoparticles in improving cellular stability and physiological efficiency of *Alstroemeria* flowers.

This superiority is likely due to increased water uptake by the stem base tissue and enhanced activity of antioxidant enzymes (such as catalase and peroxidase), which leads to a reduction in the accumulation of reactive oxygen species (ROS) and decreased lipid peroxidation; consequently, cell membrane stability is maintained, and chlorophyll degradation is slowed. This phenomenon simultaneously explains the increased petal freshness, delayed physiological senescence, and extended vase life.

Treatments containing *Plumeria* and *Calotropis* extracts both demonstrated these effects, but subtle differences between responses (especially between NP15 and NA15) are likely attributable to the distinct bioactive compounds present in the initial nanoparticle synthesis extract. The phenolic and flavonoid compounds of *Plumeria* have resulted in greater stability and more uniform distribution of nanoparticles in the solution, whereas the alkaloidal compounds of *Calotropis*, by directly influencing plant defense enzyme activities, have elicited a stronger response in antioxidant traits.

At concentrations higher than 15 ml/L, a slight decrease in anthocyanin and reducing sugars was observed, which is probably related to mild silver ion toxicity effects or osmotic imbalance. This finding correlates well with nanoparticle data from TEM and zeta potential, as high-concentration samples showed a greater tendency for particle aggregation and reduced ion exchange capacity.

In summary, the results indicate that green silver nanoparticles synthesized from plant extracts not only extend vase life through antimicrobial effects and maintenance of flower water balance but also multi-facetedly improve the physiological performance of *Alstroemeria* flowers by activating antioxidant pathways and genetically regulating stress responses. This analysis provides a comprehensive biological interpretation of the physiological, biochemical, and correlation heatmap data, clarifying the reasons for performance differences between the two types of nanoparticles.

## Discussion

The results indicated that the use of high concentrations of silver nanoparticles alone may negatively affect flower longevity, while green nanoparticles at appropriate doses can have beneficial effects. Additionally, the present study showed that using sucrose alone does not have a positive effect on flower longevity, which may be due to providing favorable conditions for microbial growth. Increased bacterial growth in preservative solutions leads to blockage of stem vessels in cut flowers and reduces water uptake, ultimately shortening vase life^31,32^. Silver nanoparticles (AgNPs) exhibit potent antimicrobial properties due to their high surface-to-volume ratio, enabling effective interaction with microbial cells even at low concentrations^33^. Costa et al.^34^ reported that silver nanoparticles, by affecting ethylene activity in plant tissues, prevent microbial growth and delay senescence.

Regarding extracts, researchers believe that plant extracts, due to their abundant antimicrobial compounds, can reduce bacterial counts in vase solutions, thereby preventing vascular blockage and increasing vase life^35^. The use of green *Plumeria* nanoparticles at low concentrations prevents premature flower senescence by reducing oxidative stress and preventing excessive production of reactive oxygen species (ROS). Ma et al.^36^ reported that pretreatment of cut carnation flowers with green nanoparticles effectively reduced bacterial-induced xylem vessel blockage, leading to increased vase life and postharvest quality. Green-synthesized silver nanoparticles (AgNPs) using Hibiscus rosa-sinensis flower extract exhibit significant antimicrobial activity, effectively inhibiting microbial growth in vase solutions and extending the vase life of cut flowers^37^. In these studies, green nanoparticles increased postharvest longevity by maintaining cell membrane integrity, inhibiting microorganism growth in the pulse solution, and reducing flower respiration.

The use of green nanoparticles in the NP10 treatment led to optimal flower bud opening. These nanoparticles, synthesized with *Plumeria* plant extract, contain antioxidant compounds including flavonoids, phenols, and terpenes, which have antimicrobial activity and regulate oxidative stress in plant cells. Green-synthesized nanoparticles, particularly those derived from *Plumeria rubra* flower extract, exhibit potent antimicrobial and antioxidant properties due to the presence of bioactive compounds such as flavonoids, phenols, and terpenoids, which enhance water retention and improve flower bud development^38^. On the other hand, the N20 treatment, which included chemically synthesized silver nanoparticles at high concentration, caused toxic effects. Nanoparticles at high concentrations can lead to excessive production of reactive oxygen species (ROS), resulting in cellular damage, reduced activity of antioxidant enzymes, membrane degradation, premature flower senescence, and decreased flower bud opening^39^. Combined treatments (NP and NA at different concentrations) along with sucrose also showed relatively good performance, but the best opening was observed in NP10, indicating the importance of optimal concentration and biological source of the nanoparticle.

Overall, the results indicated that treatments containing biologically synthesized silver nanoparticles with plant extracts performed better in increasing flower diameter compared to other treatments. This superior performance is likely due to the combined physiological effects of biological nanoparticles, such as stimulating cell growth, regulating water exchange, and delaying aging mechanisms. Additionally, the comparison of green silver nanoparticles with chemically synthesized silver nanoparticles showed that green nanoparticles combined with sucrose generally had higher efficiency. These findings are consistent with research results on *Gerbera*^40^, where green silver nanoparticles significantly improved ornamental traits.

Silver nanoparticles synthesized with *calotropis* (NA) showed weaker performance at some concentrations compared to other treatments. Treatment with plant-mediated silver nanoparticles significantly increased vase life and water uptake in cut Gladiolus spikes by reducing microbial blockage and enhancing solution absorption^41^. The use of nanoparticles synthesized from plant sources, due to their antioxidant properties and nano-structure, can improve water uptake and reduce flower senescence.

Results indicate that the application of biological nanoparticles, especially silver nanoparticles synthesized with *Plumeria* (NP) at an appropriate concentration, can lead to the retention or increase of water in flower tissues and consequently increase fresh weight. This can be attributed to improved water uptake by nanoparticles, reduced transpiration, increased cell membrane strength, and decreased microbial growth. The present results align with findings by Juthee et al.^42^ in *Gerbera*, which showed that silver nanoparticles increased water uptake and consequently fresh flower weight. On the other hand, treatments such as sucrose (S) alone or chemically synthesized silver nanoparticles (N10 and N15), although effective on some days, were less effective in maintaining fresh weight over the long term. This may be due to differences in the interaction of biological nanoparticles with plant cells and regulation of plant water^43^.

It was observed that most nanoparticle treatments increased soluble solids compared to the control. These results are likely related to the effect of nanoparticles in improving photosynthetic performance and increasing the accumulation of soluble sugars in flower tissues. According to Nayab and Akhtar^44^, Green-synthesized silver nanoparticles (from Eucalyptus leaf extract) used as an edible coating reduced ripening and maintained physicochemical parameters of banana during storage — treated fruits showed less change in total soluble solids (TSS) compared with untreated control, indicating that biogenic AgNP coatings can help preserve soluble solids and postharvest quality, which is consistent with our findings. A noteworthy point in this study is the performance of the sucrose-only treatment (S) compared to combined treatments. In most cases, combined treatments such as NP, NA, and N along with sucrose had a greater effect on increasing soluble solids, which may be due to the complementary role of nanoparticles in sugar uptake and transport in flowers. Conversely, the inclusion of sucrose in vase solutions enhanced water uptake, fresh weight, and vase life of cut Gladiolus flowers, but these improvements were significantly augmented when combined with plant growth regulators such as GA₃ and BA^45^. Overall, it appears that combining sucrose with nanoparticles, especially plant-based nanoparticles, can be an effective strategy to improve the physiological quality of ornamental flowers and performs better than using sucrose alone.

Ion leakage is an important indicator for assessing cell membrane health, and its increase indicates membrane structure damage caused by environmental stresses or toxicity. On the third day, the sucrose treatment significantly reduced ion leakage and had the lowest ion leakage among all treatments. The increase in ion leakage in treatments containing silver nanoparticles and green nanoparticles may be due to higher toxicity of free silver ions at high concentrations or the lack of sufficient antioxidant compounds in chemically synthesized nanoparticles. Gratao et al.^46^ also noted that heavy metal and nanoparticle toxicity can cause lipid oxidation and cell membrane damage, leading to increased ion leakage. Biosynthesized silver nanoparticles from *Nepeta bracteata* flowers can maintain membrane integrity by modulating antioxidant enzyme activity and lipid peroxidation in cancer cells^47^.

Results related to total chlorophyll showed a decreasing trend from the third to the ninth day in most treatments. This decline may be due to physiological stresses caused by the treatments, toxicity effects of nanoparticles at high concentrations, or changes in chloroplast structure^48^. Regarding plant-synthesized nanoparticles (biological nanoparticles), despite expectations of protective effects, a decrease in chlorophyll content was also observed in some cases such as NA10. This may indicate plant sensitivity to active compounds in the plant extract or to particle size and density, which negatively affect photosynthesis. However, these effects were more pronounced in the early days and by the ninth day, the reduction in chlorophyll had stabilized in most treatments. These findings show that nanoparticle type, concentration, and plant species are determining factors in the final effect on chlorophyll.

The pattern of changes shows that green nanoparticles (especially NP15) had a prominent effect on preserving flower pigments in the early days, whereas chemical nanoparticles (particularly N20 on the sixth day) showed greater effects over time. These results reflect the potential of plant compounds in maintaining the visual quality of flowers during early storage stages, likely due to antioxidant compounds present in the plant extract. Silver nanoparticles synthesized using plant extracts, acting as reducing and stabilizing agents, can stabilize the nanoparticle structure and, through the functional groups of anthocyanins, enhance colloidal stability^49^. In general, green (plant-based) nanoparticles are more effective in the short term and exhibit higher antioxidant activity, while chemical nanoparticles are more stable in the long term. Therefore, it is recommended in commercial storage programs to use a phased combination of both approaches to optimize both visual quality and flower longevity.

Comparison between green (biogenic) and chemical nanoparticles showed that green nanoparticles at low concentrations (NA10 and NA15) had effects similar to or slightly less than chemical nanoparticles on reducing sugar. This may indicate greater safety and better environmental compatibility of this type of nanoparticles. Green-synthesized gold nanoparticles induce adaptation in photosynthetic responses, sugar and nitrogen metabolism, and seed yield of salt-stressed mustard plants^50^.

Decrease in ascorbate peroxidase activity from day three to day nine indicates an increase in oxidative stress caused by flower senescence, which has also been reported in previous studies^51^. Additionally, according to Fig. 11, the synergistic effect of sucrose and silver nanoparticles was also observed in maintaining enzyme activity, which has been confirmed by other researchers as well^52^. Catalase enzyme activity, which is a key enzyme in combating oxidative stress, was higher in treatments containing silver nanoparticles synthesized from *calotropis* plant extract (NA) compared to other treatments. This may be due to the presence of stable antioxidant compounds in the *calotropis* extract, which play a protective and stabilizing role in the green nanoparticle synthesis process. Interaction of silver nanoparticles with catalase shows that AgNPs can form surface complexes with CAT, leading to conformational changes and modulation of its enzymatic activity—either inhibiting or altering it depending on the context^53^.

Comparing chemical silver nanoparticles (N) with plant-based nanoparticles (NP and NA), results showed that NA treatments maintained higher catalase activity on most days. Silver nanoparticles synthesized using plant extracts exhibit antioxidant properties comparable to or exceeding those of chemically synthesized nanoparticles, while demonstrating lower biological toxicity^54^. Conversely, treatments containing NP, especially NP15, showed the lowest enzyme activity on several days, which may be due to the presence of unstable phenolic compounds in the *Plumeria* extract. Overall, the significant increase in catalase activity in treatments containing *calotropis* nanoparticles on days three and six indicates that biological nanoparticles can play an effective role in combating oxidative damage in flower cells and are very practical in postharvest management. Chitosan nanoparticles significantly enhanced catalase and other antioxidant enzyme activities in cut *Rosa hybrida* petals, resulting in prolonged vase life and reduced oxidative damage^55^.

The observed differences in the effectiveness of silver nanoparticles synthesized using *Plumeria rubra* and *Calotropis procera* extracts can be attributed to variations in their physicochemical properties and their distinct interactions with the plant’s biological system. Silver nanoparticles produced with *Calotropis procera* extract, due to their smaller size and more negative zeta potential (as reported in the characterization section), exhibited higher colloidal stability. These physical characteristics facilitated more effective nanoparticle uptake, resulting in improved control of microbial contamination in the vase solution and, consequently, enhanced vase life. In contrast, silver nanoparticles synthesized with *Plumeria rubra* extract, at optimal doses (NP15), showed greater capacity to reduce the activity of enzymes involved in oxidative stress and to maintain chlorophyll levels in leaves. This superior performance in preserving quality traits suggests a stronger interaction of *Plumeria* nanoparticles with the plant’s internal pathways to reduce reactive oxygen species (ROS) production and strengthen defensive mechanisms. Therefore, both types of nanoparticles exert their positive effects through different mechanisms—microbial control by *Calotropis* and oxidative stress management by *Plumeria*—both of which are essential for extending the vase life of *Alstroemeia* cut flowers.

## Conclusion

The results of this study demonstrated that the application of biosynthesized silver nanoparticles from *Plumeria* rubra (NP) and Calotropis procera (NA) plant extracts had a significant impact on improving the physiological, biochemical characteristics, and vase life of cut *Alstroemeria* flowers. Among the treatments, NP10 was identified as the most effective treatment for significantly increasing vase life and maintaining overall postharvest quality. While NP15 and NA15 also showed superior performance in improving certain physiological and biochemical traits. The findings indicate that bionanoparticles, especially NP10, contribute to a longer vase life by enhancing physiological quality, preserving tissue structure, and promoting flower opening. As bionanoparticles are generally more biocompatible, stable, and safer than chemically synthesized nanoparticles, their use in postharvest management of ornamental flowers is recommended as a sustainable and environmentally friendly approach.

## Data Availability

Data is provided within the manuscript.
